# Investigating the role of environmental factors in the French highly pathogenic avian influenza epizootic in 2022–2023

**DOI:** 10.3389/fvets.2025.1541019

**Published:** 2025-06-13

**Authors:** Maryem Ben Salem, Mathieu Andraud, Stéphanie Bougeard, Virginie Allain, Morgane Salines, Rodolphe Thomas, Audrey Schmitz, Legrand Saint-Cyr, Karine Fiore, Sophie Le Bouquin, Axelle Scoizec

**Affiliations:** ^1^Epidemiology, Animal Health and Welfare Unit (EpiSaBe), Ploufragan-Plouzané-Niort Laboratory, ANSES, Ploufragan, France; ^2^Avian and Rabbit Virology, Immunology and Parasitology Unit (VIPAC) and French National Reference Laboratory for Avian Influenza, Ploufragan-Plouzané-Niort Laboratory, ANSES, Ploufragan, France; ^3^Social Sciences, Economics and Society Department (DISSES), ANSES, Maisons-Alfort, France

**Keywords:** poultry, epidemiology, clustering, random forest, machine learning, classification

## Abstract

**Introduction:**

The recurring epizootics of highly pathogenic avian influenza (HPAI) in France have been associated with changes in the epidemiological landscape, such as higher frequency of detections in wild birds and introductions into backyard farms. This highlights the need for a deeper understanding of the factors that drive the spread of HPAI, particularly environmental ones, which, unlike other factors, are still understudied.

**Methods:**

In this study, we examined various farm and environmental variables around the 2022–2023 outbreak sites in France to unravel potential common traits among detected outbreaks. From August 2022 to March 2023, 397 poultry farms were infected, including different species and production types. For each outbreak, the farm characteristics and variables related with their direct environment within a 2 km radius were collected. Based on the Gower distance, accounting for qualitative and quantitative variable, clusters were identified using k-medoid partitioning algorithm. A random forest analysis was further used to hierarchize the relative role of each variable in the clustering process, to assess the importance of the farm structural and environmental conditions on the outbreak occurrence. To disentangle the impact of environmental factors from intrinsic herd characteristics, the method was applied twice: first, using the whole dataset including the farm characteristics and environmental variables (first scenario); second, accounting exclusively for the environmental variables (second scenario).

**Results:**

Overall, farm variables such as farm type were crucial in the clustering process, overpassing most of the environmental factors, although the distance from “particular risk zones” and the coastline were also important. However, the clusters obtained with the second scenario that counts only for the environmental variables, remained consistent with the first scenario.

**Discussion:**

This shows a non-negligible impact of environmental conditions on the probability of viral introduction in poultry herds. This study used an innovative approach to explore how HPAI dynamics can be influenced by external factors, which could help in the design of risk zones at the national level.

## 1 Introduction

Avian influenza is an infectious disease that affects primarily wild birds and poultry (1). Depending on the characteristics of each virus strain involved, the disease can be either low or highly pathogenic (1, 2). The highly pathogenic form is extremely severe and is associated with subtypes H5 and H7 (1, 2), which were responsible for more than 31 million deaths among both poultry and wild birds and the culling of 448 million poultry birds in 114 countries between 2005 and April 2023 (1). Highly pathogenic avian influenza (HPAI) not only causes significant economic losses to the poultry industry worldwide, but also impacts the wild bird populations, and numerous mammal species. More than 48 mammal species were reported to be infected between 2020 and 2023 including minks, sea lions, dogs, cats and bears (1–6). Recent outbreaks among 695 dairy herds and a detection of a H5N1 case in pigs in the United States of America confirms the non-negligible risk of crossing the species barrier (7–9). These findings raise concerns regarding the zoonotic potential of HPAI and the risk of a new global pandemic (1, 5, 7–10).

Since its initial emergence in 1996 in Southern China, the HPAI A/Goose/Guangdong/1/1996 (GsGD) A(H5N1) virus lineage has evolved into numerous strains and spread globally (11). In 2023, it was identified in 81 countries worldwide, with the European continent being heavily affected (5, 10, 12). In 2022 alone, Europe declared 82.7% of global avian influenza infections, highlighting its crucial role as a hotspot of virus transmission (10). While severe epizootics due to recurring HPAI A(H5Nx) clade 2.3.4.4b virus introductions by wild migratory birds (13–15) have been affecting Europe since 2014, the 2021–2022 epizootic was considered the most substantial with 6,707 HPAI outbreaks, in both poultry and wild birds, followed by 2022–2023 epizootics, accounting for 5,514 outbreaks (16).

When considering the economic impact of HPAI epizootics on the European poultry sector, France stands out as one of the most affected countries, given its significant role as a producer and exporter of poultry products (15, 17–19). Over the past few years, a notable shift has been observed in the French HPAI epidemiological context. Historically, in 2016–2017 and 2020–2021, most outbreaks occurred in south-western France, a region with a distinct poultry industry, known for its high-density of duck farms, specifically involved in the production of “foie gras,” and extensive outdoor farming practices (15, 20, 21). Flock density was identified as a major explanatory factor for these epidemiological situations (15, 19). In addition, the specific structural dynamics of the “foie gras” industry include frequent duck movements between different sites (20). Access to outdoor areas further increases the risk of virus introduction into farms, due to potential direct contact with migratory and commensal wild birds visiting these open spaces (22, 23). These factors combined altogether result in a complex epidemiological landscape marked by a high risk of HPAI introduction and spread. Despite improvements in on-farm biosecurity, and reinforced legislation impairing outdoor poultry rearing during at-risk periods, major epizootics still occurred in 2021–2022 and 2022–2023, revealing important changes in HPAI dynamics in the country (24). Unlike the previous ones, these epizootics were not limited to the “foie gras” industry in south-western France, but extended for the first time to western and central-western regions, affecting a wider range of production sectors. These included other duck production (meat and breeding), turkey production, and chicken production (15). In the western region, this change in epizootic size and the variety of affected species was attributed to both the size of farms and the diversity of production types in the region (15). A striking difference compared to the past was the increase in sporadic detections of HPAI viruses on farms, in backyards, and in captive holdings all over France (24). Moreover, there was an upward trend in the number of detections in the wild bird population (24). These levels of infection and spread are unprecedented compared to previous outbreaks and indicate a substantial shift in transmission dynamics, with an endemic trend among local wildlife.

The shift in epidemiological behaviour observed in the last two epizootics, and especially the apparent endemicity of H5N1 in wild bird populations and their interaction with domestic poultry, raises questions about the potential role of environmental factors in the introduction and transmission processes in poultry production units. Despite being well discussed in the literature for their role in the introduction and spread of HPAI (22), the environmental characteristics surrounding HPAI infected farms remain poorly evaluated. In this context, using data collected during the winter HPAI epizootic of 2022–2023 in France, our study aims to investigate common traits related to farm characteristics and environmental contexts, through a clustering approach, and to analyse the relative contribution of these factors in cluster building using a random forest methodology. This article first examines potential clusters among the outbreaks following two scenarios, including different combinations of variables, and then goes on to evaluate the contribution of the different variables in the clustering process.

## 2 Materials and methods

### 2.1 HPAI poultry outbreak data

The dataset was obtained from the General Directorate for Food of the French Ministry of Agriculture, Food Sovereignty and Forestries for the period from August 2022 to June 2023. An outbreak was defined as at least one HPAI-infected bird within a poultry farm, detected through PCR methods. The following characteristics of infected farms were extracted and used in the analysis: province, municipality, geographic coordinates (World Geodetic System), farm type (commercial, backyard, and captive), species, production type, date of initial suspicion, and outbreak identification number. The different farm types, species, and production types are detailed in Table 1.

**Table 1:** Description of variables used in the analysis of HPAI outbreaks in France in 2022–2023.

Category	Variable		Description	Modalities (abbreviates) and definitions
	Analysis	Name		
HPAI poultry outbreaks variables	Scenario A	Type	Farming topology in the poultry sector	Commercial (CMMF): farms with commercial activities Backyard (BCKY): farms with no commercial activities. Birds are raised for private consumption /convenience. Backyard farms need to be registered. They are registered at provincial level if the number of birds exceeds 250; otherwise, registration at the municipality level is sufficient Captive (CPTV): birds other than chickens, turkeys, guinea fowls, ducks, geese, quails, pigeons, pheasants, and partridges that are raised or kept in captivity for breeding, meat or table egg production, supplying game birds for restocking or hunting purposes, as well as for shows, races, exhibitions, competitions, or sale
		Production type	Types and objectives of production sites	Decoy ducks (DCYD): ducks used for hunting. They serve as a decoy to attract wild birds Starter phase (STRP): The first phase of chick rearing after hatching Game birds farm (GMBF): birds raised to be released for hunting activities or species conservation Fattening (FTTN): intensive rearing phase for meat production Future laying hens (FTLH): pullets intended for laying hens Rearing period (RRNP): preparatory phase before assisted-feeding Laying hens (LYNH): adult laying hens used for egg production Assisted-feeding (GAVA): assisted-feeding phase (ducks and geese) for foie gras production Future breeders (FTRB): birds selected to be breeding stock Breeders (BRDR): Adult birds used for reproduction and genetic line continuation
		Species	Species in which avian influenza was detected	Turkey (TRKY) Duck (DUCK) *Gallus gallus* (GLLG) Multi-species without ducks or geese (MLT-SP CSWITHTW) Multi-species with ducks or geese (MLT-SP CSWITHWT) other (OTHR)
Environmental variables	Scenarios A & B	Nb. Farms	Number of poultry farms in the outbreak surroundings within a 2 km radius of each HPAI outbreak location	
		Nb. Farms +W	Number of ducks or geese farms in the outbreak surroundings within a 2 km	
		Nb. Farms - W	Number of farms not including ducks or geese in the outbreak surroundings within a 2 km	
		Dist. Case	Minimum distance from preceding outbreaks within a 7-day period for each new notification	
		Dist. Coast	Minimum distance to the coastline	
		Dist. Water Surf.	Minimum distance between each HPAI outbreak location and water surfaces	
		Nb. Water Surf.	Number of water surface features that exist within the 2 km around each HPAI outbreak location	
		Water Surf. Cover	Water coverage percentage within the 2 km buffer around each HPAI outbreak	
		Dist. Roads	Minimum distance between each HPAI outbreak location and roads	
		Nb. Roads	Number of roads that intersect the 2 km buffer around each case of HPAI	
		Road Length	Total length of roads that intersect the 2 km buffer around each case of HPAI	
		Dist. To PRZ	Category of the minimal distance between the outbreak and any particular risk zone (PRZ) municipality Three possible category: “Inside,” “Neighbouring,” and “Outside”	

Both HPAI poultry outbreak variables and environmental variables are included. For each variable, the table lists its category, analysis scenario, name (as used in the graphs), description, modalities, and abbreviates (as used in the figures and Supplementary material).

### 2.2 Environmental variables data

In our study, we refer to the factors external to the farm as “environmental factors.” Twelve variables were selected to describe the surroundings of outbreak locations (Table 1). To account for favourable wind conditions, surroundings were limited to a 2 km buffer around each outbreak location. This allows infection to spread up to twice the distance estimated in the work of Ssematimba et al. (25), where the deposition of contaminated dust was evaluated to be at its best around 0.45 km and decreased significantly beyond 1 km. Similar tendency was observed for the model predicting the probability of infection that drops sharply beyond 1 km (25).

#### 2.2.1 Poultry sector-related variables

The density and closeness of poultry farms may present a risk regarding HPAI introduction into susceptible production sites. Eight variables were considered in this category: the number of poultry farms in the outbreak surroundings within a 2 km radius of each HPAI outbreak location, the number of duck or geese farms in the outbreak surroundings within a 2 km, the number of farms not including ducks or geese in the outbreak surroundings within a 2 km, the minimum distance from preceding outbreaks within a 7-, 10-, 14-, 21- and 28-day period for each new notification. The number of poultry farms in the outbreak surroundings within a 2 km radius of each HPAI outbreak location was calculated using a database that lists all farms in France, along with their geographical locations. Following the same method, within a 2 km-radius from outbreak locations, the numbers of farms hosting and not hosting ducks or geese were counted. Finally, the minimum distance from preceding outbreaks within different time windows (7, 10, 14, 21, and 28 days) for each new notification were calculated. For the two outbreaks for which there was no detection in the previous 7 days in France, a fictitious high distance of 1,000 km, beyond the borders of France, was defined, considering introduction through wild birds or other routes, such as avian or bird trade.

#### 2.2.2 Water-related variables

Water surfaces represent favourable environments for migratory and commensal birds that could potentially interact with poultry farm animals. Four variables were considered to analyse the presence of water surfaces in the farm neighbourhood. The minimum distance to the coastline was first evaluated for each infected farm. This variable reflects the risk related to migratory and non-migratory wild shorebirds visiting or residing close to the seashore. The remaining three variables reflect the risk related to both inland visiting migratory wild birds and the commensal wild water bird population: the minimum distance between each HPAI outbreak location and water surfaces; the number of water surface features; and the water coverage percentage within the 2 km buffer around each outbreak. The BD TOPAGE^®^ database, a publicly available resource, was used for data on the hydrographic entities in the country.^1^

#### 2.2.3 Road infrastructures

Anthropogenic factors may also play a role in the transmission process. To analyse whether a relationship could be established between the outbreak occurrence and the transit of vehicles (live animals, rendering plants, etc.), three variables were considered: the minimum distance between each HPAI outbreak location and roads, the number of roads within a 2 km buffer around each HPAI outbreak location, and their total lengths. These variables were considered as a proxy for human-mediated indirect transmission through animal movements between farms (truck transit), dead bird removal, feed delivery, etc. Road infrastructure data were taken from the ROUTE 500^®^ database.^2^ For this analysis, only national and local roads were considered.

#### 2.2.4 Proximity to particular risk zones

The particular risk zones (PRZs) are zones associated with a higher risk of HPAI introduction via wild and migratory birds. Their definition is based on the presence of wetlands and migration corridors (22, 26). These zones are defined in regulations at the municipality level.^3^ To explore the link between proximity to a particular risk zone (PRZ) municipality and HPAI outbreaks, we calculated the minimum distance between each outbreak and PRZ municipalities. We defined three categories to classify the outbreaks: “Inside” for outbreaks located in a PRZ municipality; “Neighbouring” for outbreaks at a distance of less than 2 km from a PRZ municipality; and “Outside” for outbreaks more than 2 km away from a PRZ municipality.

### 2.3 Description of the epizootic

To describe the first wave of the 2022–2023 epizootic in terms of number of outbreaks, the distribution of outbreak type, and affected species, a descriptive analysis was conducted. All analyses were carried out using Rstudio, version R-4.2.2 (27). French boundaries and administrative regions (level 1) were downloaded from the GADM^4^ database.

### 2.4 Cluster analysis

To identify potential clusters of HPAI outbreaks having similar characteristics and to investigate their association with the different variables, particularly environmental ones, a cluster analysis using a two-step approach was followed.

#### 2.4.1 Cluster identification

The Gower distance method was applied to measure similarity and dissimilarity between observations in our mixed dataset (i.e., numeric, nominal, ordinal) (28). Afterwards, the partitioning around medoids method was employed to distribute the dataset in K distinct clusters, where the groups of individuals share common patterns (29). The number K of clusters was defined through visual inspection of the silhouette plot that evaluates the appreciation of the closeness of each point to a neighbouring cluster (30). To visualise the potential clusters, the t-distributed stochastic neighbour embedding (t-SNE) method was used as a technique to represent complex data in a lower-dimensional space (31).

#### 2.4.2 Variable contribution in the clustering process

The random forest algorithm was used to identify explanatory variables that best discriminate clusters (considered as categorical dependent variable) (32, 33). This choice of a non-parametric learning method was driven by the different formats of our explicative variables (i.e., numeric, nominal, and ordinal) (32). The dataset was split into training and test sets, with 60% allocated to the training set and 40% to the test set. A random forest model was trained on the training set to assess the importance of different variables. Hyperparameters were kept at their default values, as their variation did not significantly affect the random forest outcomes.

This algorithm builds a number of decision trees (by default 500 trees are used) based on a bootstrap sample of the training set [approximately 63% of the data points are used to train each tree, leaving the remaining 37% for out-of-bag (OOB) evaluation] and a selection of variables and observations (34, 35). By using a majority voting system, it then aggregates the predictions of all trees to determine the class of the next example (34). Model performance was evaluated using OOB error estimates and predictions on the test set using a confusion matrix. The OOB error is a measure that evaluates global model quality (34). Once a tree is trained on its bootstrap sample (the 63% of data), the associated 37% OOB data is used to test the tree. Predictions made on the OOB data provide an independent evaluation of the tree’s performance, without using the data it was trained on. It indicates the percentage of well-classified observations among the OOB samples (32). The mean decrease in accuracy represents the average reduction in the model’s accuracy resulting from the removal of a given variable and is generally used to hierarchize the importance of the different factors (36). A substantial decrease in the accuracy error indicates a more important variable in the correct prediction of classes (34, 36, 37).

The entire cluster analysis process, including both cluster identification and the evaluation of variable participation in the clustering, was done based on two scenarios. The first scenario, hereafter, referred to as scenario A, included all variables (i.e., intrinsic farm characteristics along with environmental variables) to design potential clusters of infected farms sharing similar characteristics. The second scenario (scenario B), the process was repeated focusing exclusively on environmental variables to evaluate whether they could be discriminant to identify potential critical zones of transmission. This entire process aimed to evaluate the specific role of environmental factors in the clustering (32).

## 3 Results

### 3.1 Description of the epizootic

**Figure 1:**
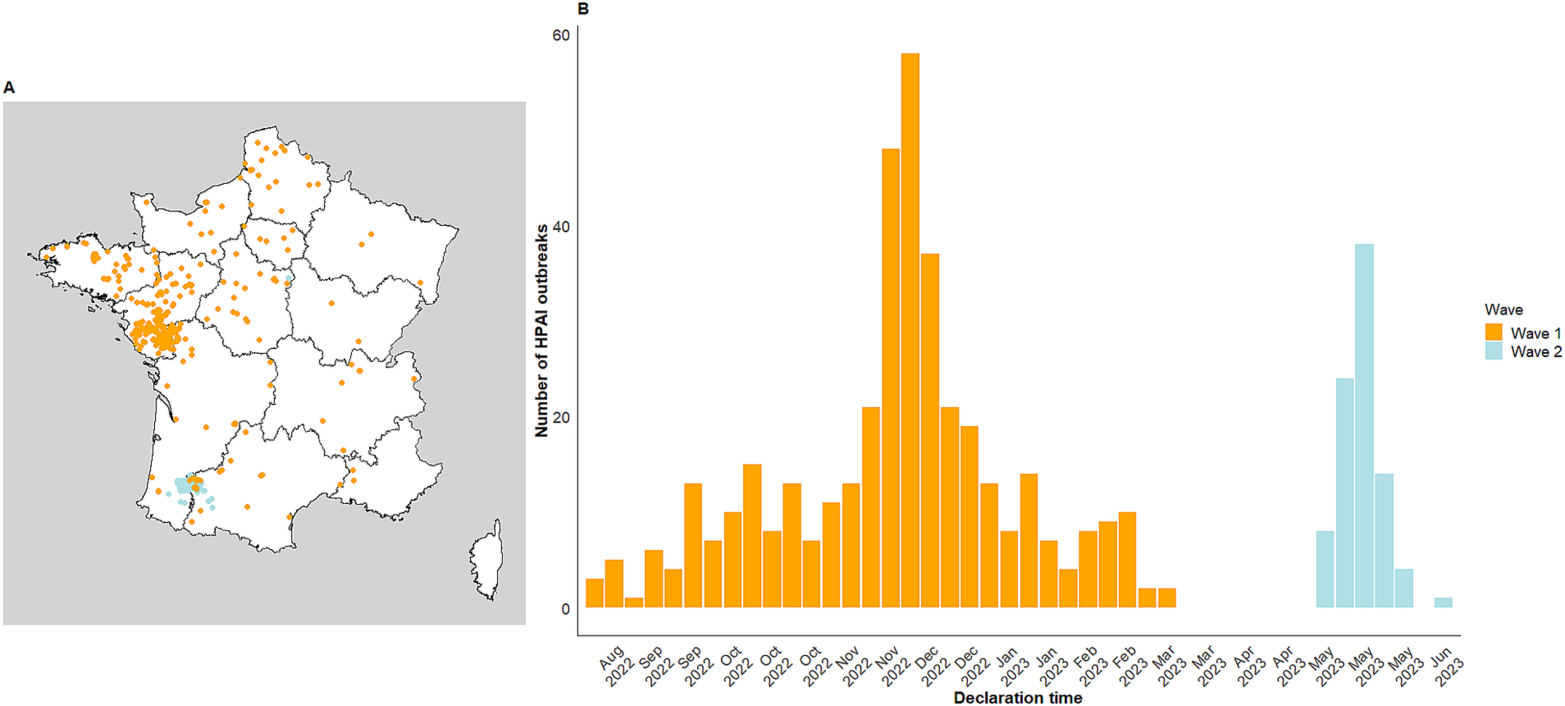
Distribution of HPAI outbreaks in poultry in France from August 2022 to June 2023 illustrated as follows: **(A)** Geographical distribution; **(B)** Temporal distribution. Two waves can be seen: from August 2022 to March 2023 (397 cases, orange) and from May to June 2023 (89 cases, light blue).

The 2022–2023 epizootic was characterised by two waves over two distinct periods. The first wave affected all of mainland France, with a high number of outbreaks in western France (Figure 1, orange). This first wave was followed by a separate, second wave that rapidly and quite intensively affected a restricted area in south-western France (Figure 1, light blue). A total number of 486 outbreaks were reported during the epizootic as a whole. Given the differences between the two waves, this work will focus on the first wave starting from August 2022 to March 2023. During the first wave, 397 outbreaks of HPAI were reported in France, with a peak in early December 2022 (Figure 1B, orange). A high number of outbreaks was observed in north-western France (Figure 1A, orange). In this analysis, 396 outbreaks were taken into account as one observation was incomplete (one production type not documented). The outbreaks mainly affected commercial farms (312 farms representing 78.7%), but also backyard flocks (69 backyard flocks representing 17.4%). The remaining outbreaks concerned captive holdings (15 captive holdings representing 3.8%). Among the affected species, ducks were the most commonly reported species (186 representing 46.96%), followed by *Gallus gallus*—referring to broilers and laying hens—(89 representing 22.47%). Multi-species farms with ducks or geese (47 representing 11.8%) and turkeys (46 representing 11.6%) were also affected.

### 3.2 Cluster analysis

#### 3.2.1 Cluster identification

**Figure 2:**
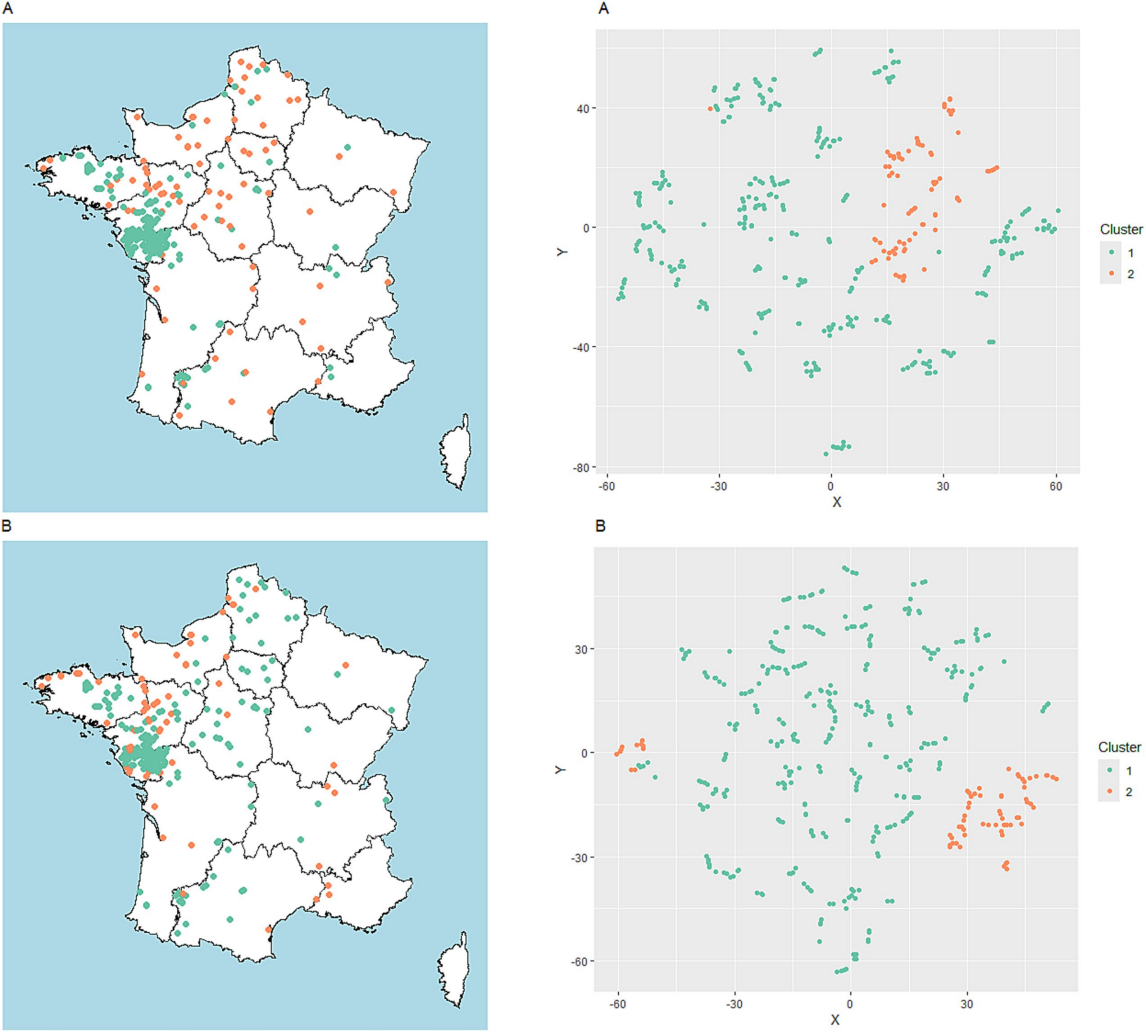
Geographical distribution of clusters and associated cluster plots applied to HPAI outbreaks data in poultry in France from August 2022 to March 2023. For each scenario (**A**: farm characteristics and environmental variables; **B**: environmental variables), the left side of the figure represents the geographical distribution of the clusters observed using the k-medoids clustering method. The right side represents the cluster plot of k-medoids obtained using the t-distributed stochastic neighbour embedding (t-SNE) method applied to HPAI outbreaks data.

In both scenario A and B, the outbreaks were distributed in two clusters sharing more than 75% similarity (300 out of 396 outbreaks were classified similarly) (Figures 2A,B; Supplementary Figure 1).

**Figure 3:**
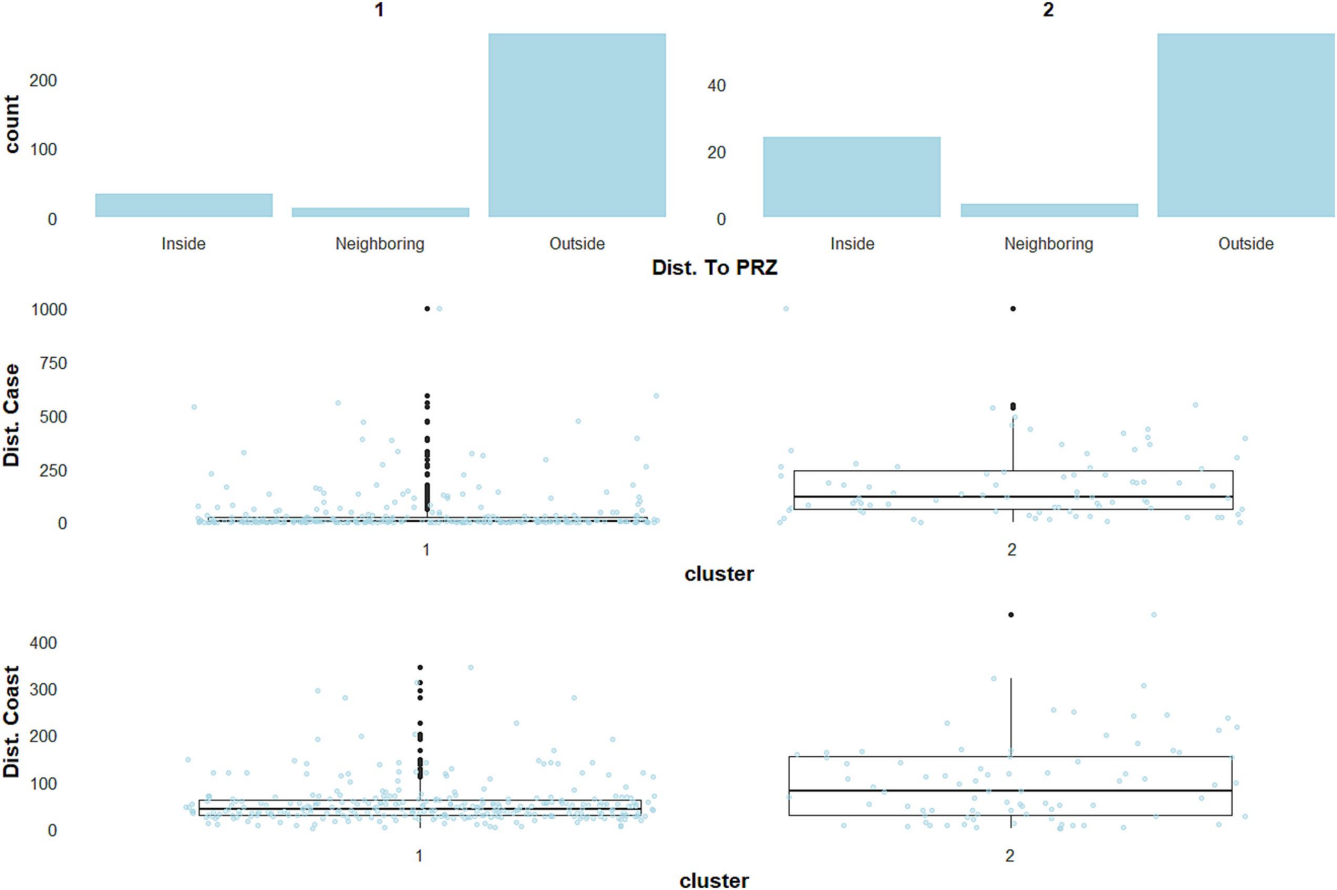
Descriptive statistics for three environmental variables in scenario A, reflecting the category of the distance to particular risk zones (Dist. To PRZ), the distance between outbreaks within 7 days (Dist. Case), and the distance between cases and coastline (Dist. Coast).

When taking into account both farm characteristics and environmental variables (scenario A), the major cluster, hereafter referred to as cluster A1, included 313 outbreaks and was essentially composed of commercial duck production farms, followed by *Gallus gallus* and turkey production sites (Figure 2A, blue-green dots; Supplementary Figure 2). The second cluster referred to as cluster A2, (Figure 2A, dark orange dots; Supplementary Figure 2) included 83 outbreaks mainly in backyard flocks hosting multiple species, including ducks or geese. Analysis of the environmental variables for each cluster revealed specific features. For example, the closeness in time and space between cases was found to be different: distances between outbreaks within 7 days were lower in cluster A1 than in cluster A2 (Figure 3). Only distances between outbreaks within 7 days were thereafter considered, as the results for other time-windows tested were very stable over longer durations (data not shown).

**Figure 4:**
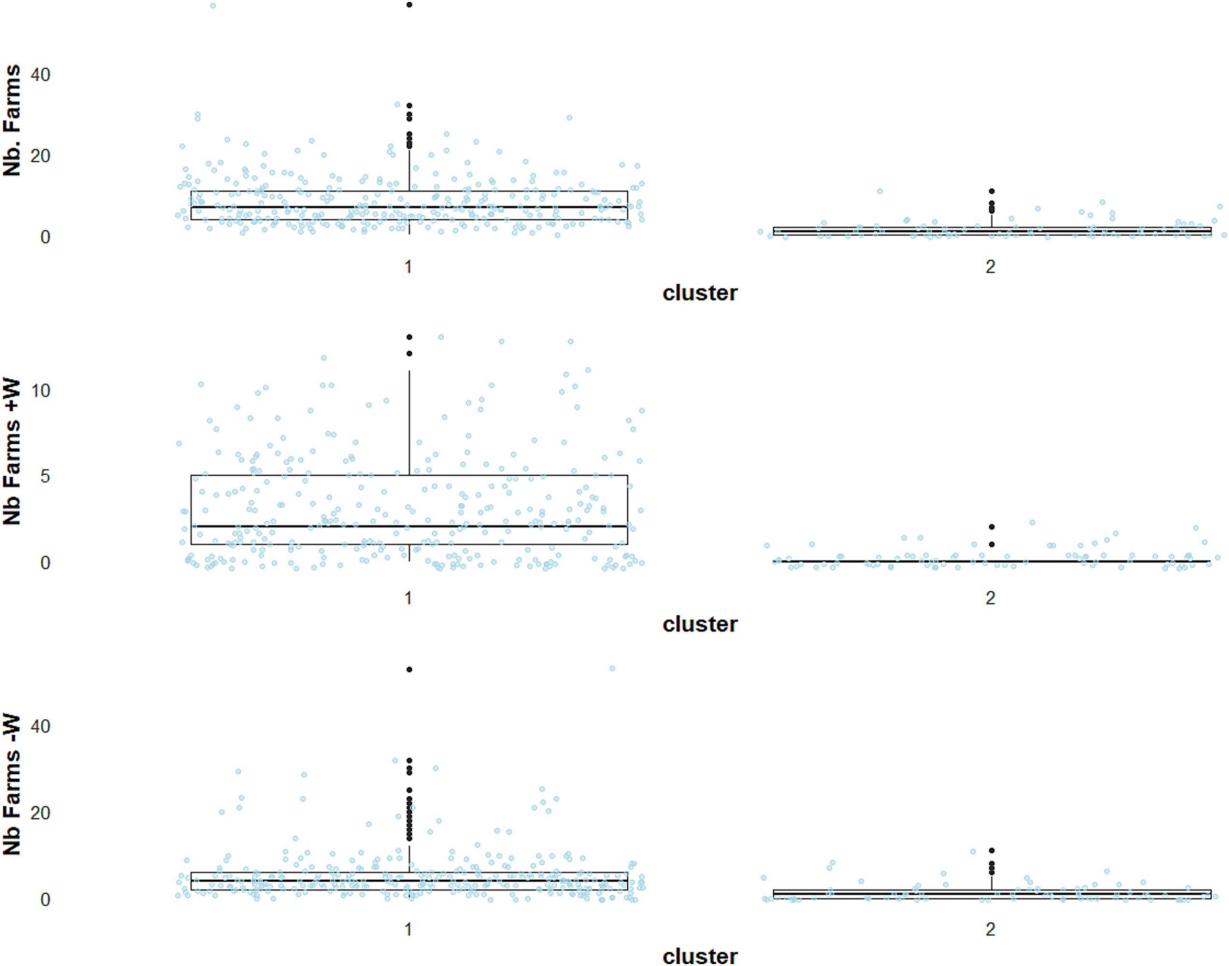
Descriptive statistics for three environmental variables in scenario A, reflecting the category of the number of neighbouring farms within a 2 km (Nb. Farms), the number of neighbouring farms hosting ducks or geese (Nb. Farms +W), and the number of neighbouring farms not hosting ducks or geese (Nb. Farms −W).

Moreover, the number of neighbouring farms within a 2 km radius was also higher, reaching a maximum of 57 farms in cluster A1, while at most 11 farms were identified in cluster A2 (Figure 4).

This difference was higher when considering the presence of ducks or geese farms in the neighbourhood. Interestingly, 85% of the outbreaks in cluster A1 were located outside PRZ municipalities, with just 10.9% of the outbreaks inside PRZ municipalities (Figure 3). Cluster A2 consisted exclusively in sporadic cases, 33.7% of which were located within or in the neighbourhood of PRZ (Figure 3; Supplementary Figure 2). Additional information about the environmental variables for scenario A is shown in Supplementary Figure 3.

**Figure 5:**
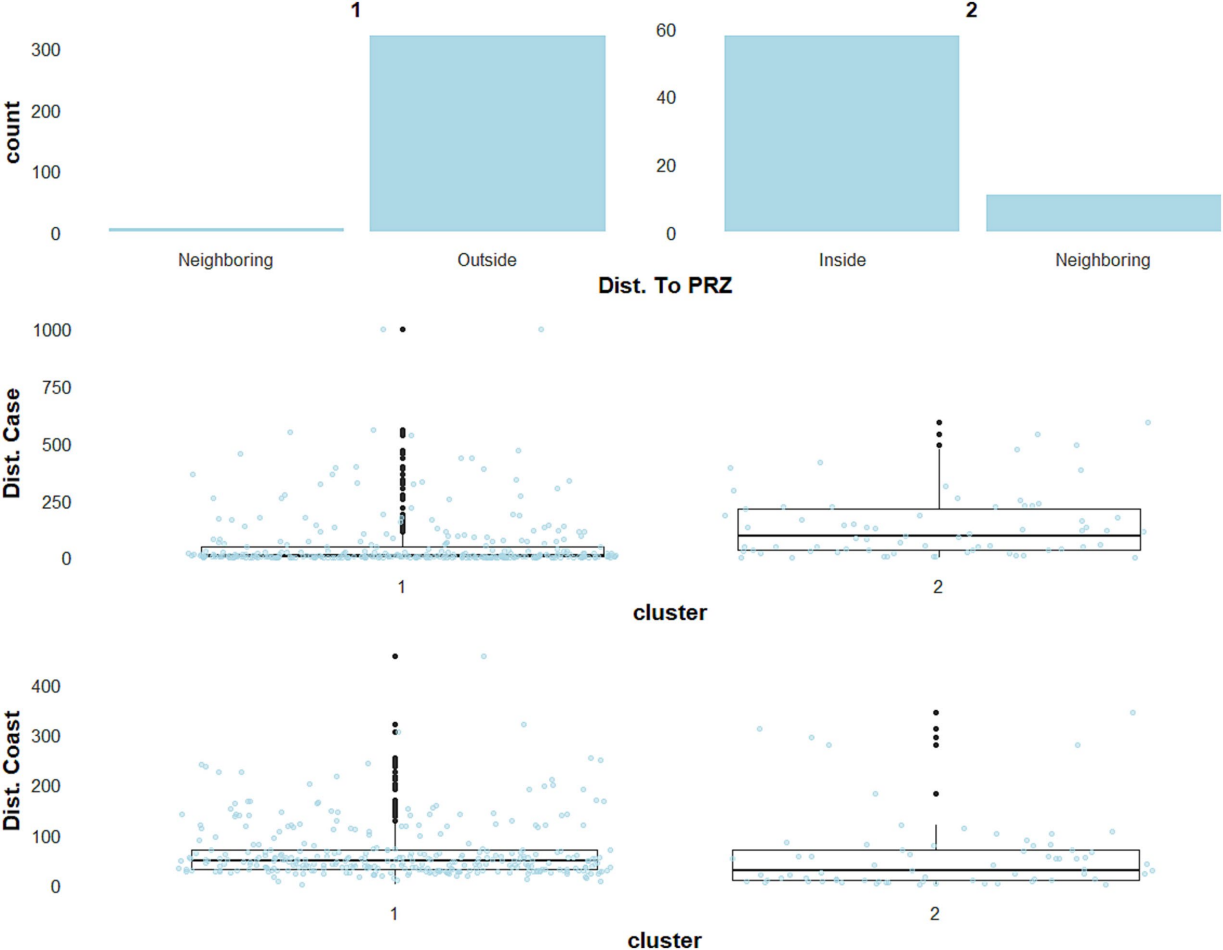
Descriptive statistics for three environmental variables in scenario B, reflecting the category of the distance to particular risk zones (Dist. To PRZ), the distance between outbreaks within 7 days (Dist. Case), and the distance between cases and coastline (Dist. Coast).

In scenario B, despite the absence of farm characteristic variables, two distinct clusters were also found (Supplementary Figure 1). The results were highly consistent with scenario A in terms of descriptive environmental variables. However, a difference was found concerning proximity to PRZs. While cluster A2 was composed of farms located inside and outside PRZ municipalities in scenario A, the results of scenario B showed a strong dichotomy in the clustering process based on this feature (Figure 5).

**Figure 6:**
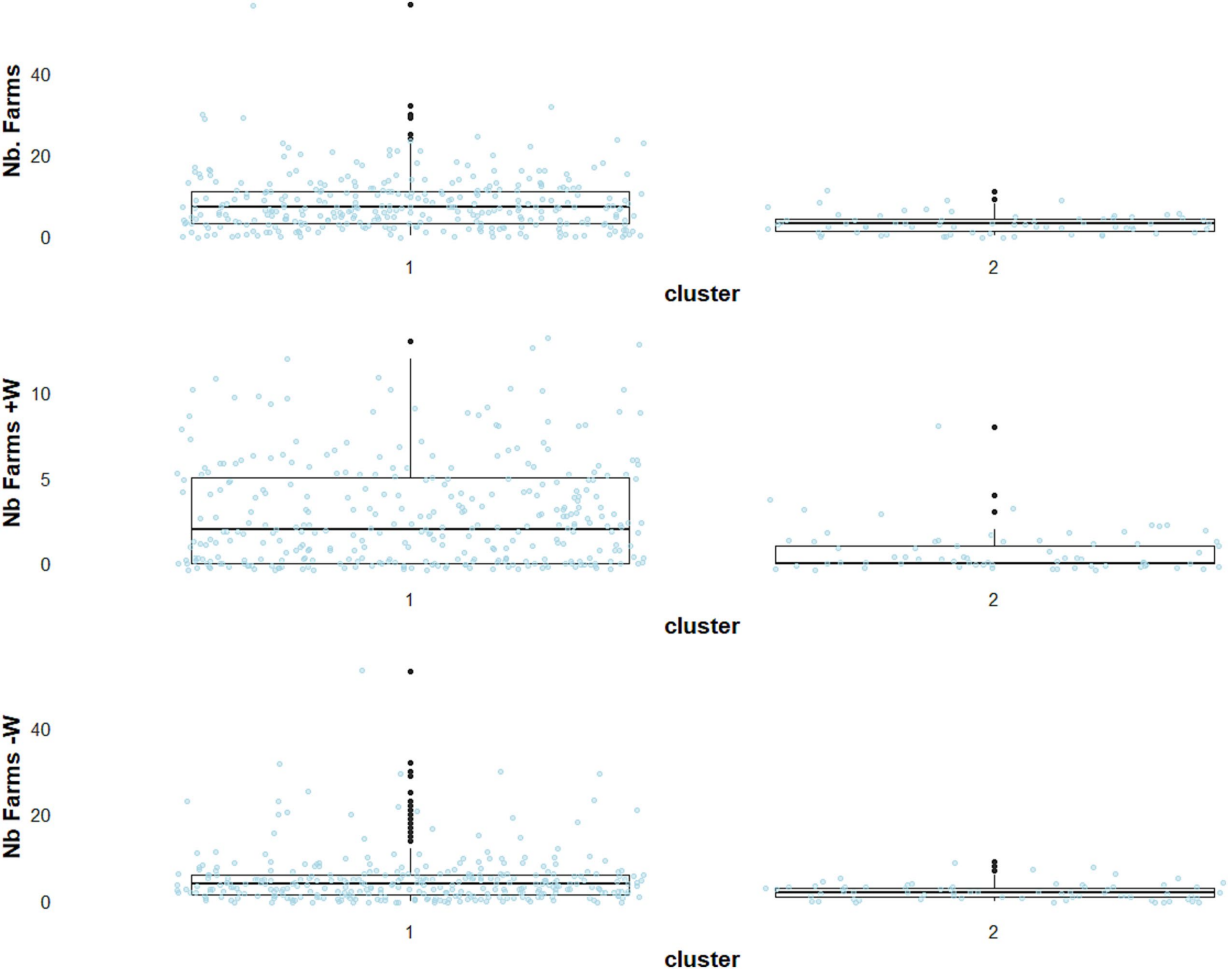
Descriptive statistics for three environmental variables in scenario B, reflecting the category of the number of neighbouring farms within a 2 km (Nb. Farms), the number of neighbouring farms hosting ducks or geese (Nb. Farms +W), and the number of neighbouring farms not hosting ducks or geese (Nb. Farms −W).

Cases in the main cluster, referred to as cluster B1, were located almost exclusively outside PRZ municipalities (98.2%), while the second cluster, referred to as cluster B2, was mainly in PRZ municipalities (84%) (Figure 5). The cluster B1 accounted for 327 outbreaks (Figure 2B, blue-green), with 83% matching with cluster A1 in scenario A. Again, outbreaks from the main cluster (B1) were relatively close to each other, with a median minimum distance of 7.2 km for successive outbreaks over 7 days (Figure 5). Cluster B2 consisted exclusively in outbreaks located within (84%) or in the neighbourhood of PRZ, with sporadic geographically separated outbreaks (Figure 5) (average distance 92 km). The number of farms in the surroundings of outbreak locations remained higher in cluster B1, as in scenario A, but the difference tended to be slighter, with a median of seven farms in cluster B1 and three in cluster B2 (Figure 6).

The outbreaks in cluster B2 (Figure 2, dark orange) were closer to the coastline, with a median distance of 29 km (Figure 5). Additional information about the environmental variables for scenario B is shown in Supplementary Figure 4.

#### 3.2.2 Variable contribution in the clustering process

**Figure 7:**
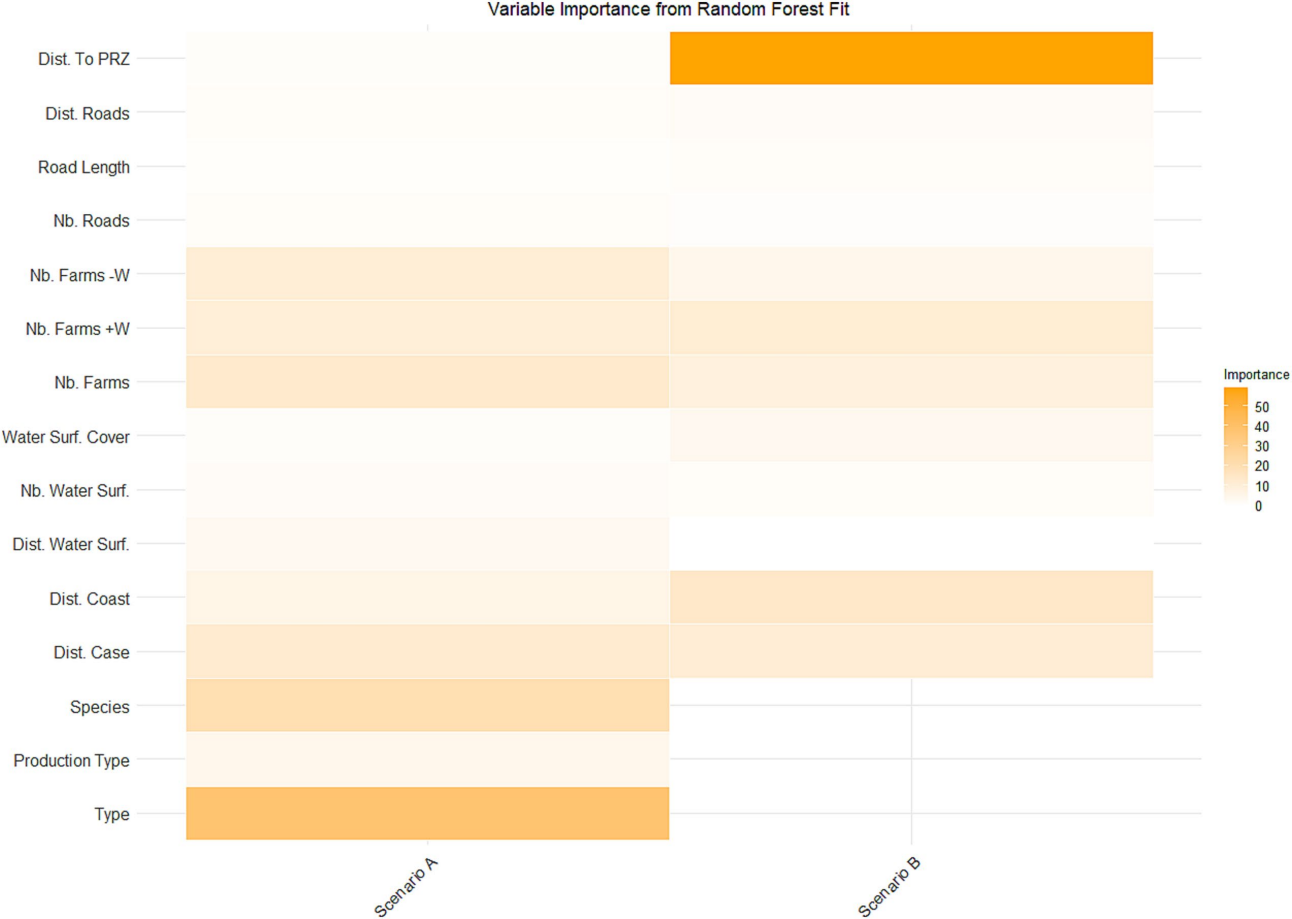
Heat map of the importance of variables for each scenario (scenario **A**: farm characteristics and environmental variables; Scenario **B**: environmental variables) using the mean decrease in accuracy. The different variables labels are detailed in Table 1.

Out of bag (OOB) errors obtained for both scenarios A and B indicated good quality models and satisfactory predictive capacity (OOB <2%). For scenario A, the study of variable importance through the mean decrease in accuracy highlighted the importance of farm type. In decreasing order of importance, species, number of neighbouring farms, and minimum distance between successive outbreaks over 7 days were also important (Figure 7). For scenario B, the mean decrease in accuracy confirmed that the distance to PRZ municipalities was the most important variable (Figure 7). The impact of distance to coastline, number of neighbouring farms housing ducks or geese, and the minimum distance between successive outbreaks over 7 days was also demonstrated, but to a lesser extent. For both scenarios A and B, variables related to roads seem to be among the least important (Figure 7).

## 4 Discussion and conclusion

Our study described the HPAI outbreaks between August 2022 and March 2023, with an emphasis on farm characteristics and environmental features in the surroundings of outbreak locations. To this end, two nested hypotheses were designed and analysed sequentially, through the development of an original approach. The objective was to draw common traits from individual data with no prior knowledge of their interactions or epidemiological relationships, accounting for both qualitative and quantitative variables. Clusters were identified using the Gower distance and k-medoid partitioning algorithm in order to qualify infected farms according to their intrinsic and environmental characteristics. A random forest analysis was used in a second phase to rank the relative role of each variable in the clustering process.

In the present study, two scenarios were presented. The first includes all available variables, i.e., related with the farm characteristics and their direct environment. The second focuses exclusively on environmental variables, excluding farm characteristics. It came out a third scenario, with only farm characteristics in, would also be relevant. However, the analysis (data not shown) suggested eight clusters as the optimal number of classes, with no rationale behind. This was likely due to an overfitting of data, given the three explanatory variables, as confirmed by forcing the number of clusters to 2 in the clustering algorithm. Indeed, such forcing led to an overlap of the clusters with the first scenario including all the variables. This confirms the importance of the farm types and species in the infectious process as evidenced by the random forest analysis.

Adding environmental variables to the analysis allowed the algorithm to identify the optimal number of clusters as 2, with different geographical expansion. One cluster was limited to the north-western region, affecting different species, including ducks, *Gallus gallus*, and turkeys. This region already experienced an epizootic in 2021–2022, but with different timing since a first wave was described beforehand in the south-western region (9), resulting in a high risk period across the country. In 2022–2023, only sporadic and isolated cases were described in the poultry sector before the introduction in the north-western region. Overall, more sporadic introductions took place during the 2022–2023 Avian Influenza season, probably due to higher HPAI prevalence among wild birds. In south-western France, the significant change in duck farm density right in the heart of duck production zone, under the framework of the “plan Adour”—consisting in preventively emptying farms during at-risk period in highly densely duck production areas—could explain why this region did not experience large outbreaks in winter 2022–2023 (15, 24).

Another particularity of the two last epizootics is the diversity of affected species, with a higher proportion of infections in *Gallus gallus* when compared with historical data, where duck farms were almost exclusively infected (15). This change is probably the result of regional differences in terms of farm composition in the south-west, which has a higher concentration of duck farms, but might also reflect a shift in the epidemiological characteristics, especially in terms of transmission and receptivity among the different species (15). Accounting for environmental variables, along with farms characteristics, made it possible to identify farm types and species as the main drivers in the clustering process. Although the role of commercial duck farms in the different epizootics was very well documented (15, 20, 21, 24), this result shows that considering only the species and farm type for targeted surveillance and outbreaks management could be misleading if the farm environment is ignored. A short distance to preceding cases within a 7-day time-window was found to be pivotal to clusters definition, suggesting transmission from one to the next, which could have different origins, such as equipment sharing, common staff or visitors, or airborne transmission. This could be linked to the particular aspects of this type of production and the farms’ localisation in high-density farm regions (19, 21). The role of high-density farm regions was also documented in other European country, such as the Netherlands (38). These factors, in addition to the inherent high receptivity of ducks to HPAI viruses, increase farm susceptibility (22). The second cluster also highlighted farm type as discriminant, with backyard farms mainly involved. Poultry are also likely to be exposed to a high risk of introduction through wild birds, especially when they are located near PRZs and the coastline (22) where migratory birds are more likely to pass by. Although water surfaces were not pointed out as pivotal in our analysis, wetlands used by migratory birds could also influence the occurrence of outbreaks in both poultry and wild birds (39). These water body factors might nevertheless be underestimated, as they are mostly embedded within PRZs, making it difficult to clearly identify their actual role in the infection dynamics. The infectious pressure associated with wild birds was relatively high during 2022–2023 epizootics, which explains the increased number of cases among backyard farms (24).

Combining environmental and farm features helped to disentangle two independent groups of farms sharing similar characteristics. Could environmental variables alone be sufficient to characterise the cases? Running the analysis with these variables only, ignoring all intrinsic farm characteristics, allowed us to retrieve two clusters sharing 75% similarity with those described above. The percentage of similarity increased to 83% when considering the largest cluster in the western region. While variables indicating highly dense regions and potential connectivity, such as the number of duck farms around the outbreak site and the minimum distance between outbreaks within a period of 7 days, were unsurprisingly important, the distance to PRZs and the coastline were the most decisive ones. These two factors are signs of potential contact with wild and migratory birds, suggesting a potential role of environmental factors in the dynamics of HPAI through direct or indirect contact with wild birds (22). In our study, variables related to roads were not found to be determinant in the clustering process, as opposed to previous studies in Asia (40). This difference might be explained by the different context and road infrastructure, but also by the dissimilarity in the roads considered (40). Only national and local roads were included in our study, while Islam et al. (40) accounted for motorways, which were significantly associated with outbreaks (40). As our goal was to focus on the immediate environment of farms, limiting ourselves to a radius of 2 km, we deemed the inclusion of motorway network irrelevant. However, it could be interesting to study traffic more deeply and not just consider roads as a static variable, ignoring traffic and the volume of animals passing by. Algorithms identifying the movements of animals along the different roads could be a more accurate proxy of the importance of each road in the surroundings of the farms (41).

This study aimed to describe outbreaks beyond the typical farm characteristics, by also examining their close environment. We successfully identified environmental factors as being able to discriminate between two groups of cases in France between August 2022 and March 2023. The data used in the present study consisted uniquely in farm and environmental characteristics, along with outbreak detection times. These data did not allow to distinguish primary and secondary outbreaks. Combining epidemiological phylogenetic analyses data would be necessary for reconstruction of transmission trees (42). However, our analyses of the common traits among the different outbreaks highlighted notably the relevance of PRZs definition in relation with the risk of viral introduction on farms. Although PRZs often include coastlines, cases were also detected close to coastlines that were not in PRZs, explaining the importance of distance to coastlines in the clustering process. This finding reinforces the need to identify risk zones, which were found to be highly discriminant. Considering the national scale, and reproducing the analysis using the full dataset of poultry production sites, could help to update the definition of HPAI risk zones, according to French legislation. However, further improvements are still possible, specifically by considering the status of the neighbouring farms (empty or occupied), information which was not available at the time of this study. Including meteorological data considering wind speed and direction could also be interesting to further assess the risk of air-borne transmission.

In addition, when considering the epizootic as a whole, including outbreaks at the start of May 2023 located in the southwest and north of France, our method did not seem to be able to explain the epizootic in the same way. This finding suggests that different mechanisms were probably involved in the epizootic’s second wave, pointing out the need for additional studies. A possible way to further investigate these factors would be to use our approach by including additional variables. Some potential variables could be those collected during the epidemiological investigation of the outbreaks or variables that reflect socio-economic factors susceptible to influence disease dynamics.

While studies using clustering analyses to tackle HPAI dynamics exist, they tend to evaluate epidemiological spatio-temporal clusters using K-function, SatScan, and kernel density estimation notably in Bangladash, Denmark, and Egypt (43–45). A study focusing on risk factors and not spatio-temporal dynamics using hierarchical clustering on principal components analysis was described in South-Korea (46). Following this direction, the methodology used here proposes to also focus on the common patterns between outbreaks rather than the spatio-temporal dynamics. It represents an innovative approach in the field of veterinary epidemiology, and, to our knowledge, has only been used by Oehm et al. (37) to characterise antibody status with regard to *Fasciola hepatica* or *Ostertagia ostertagi,* two parasites affecting dairy cows. This methodology seems promising as it offers a wide spectrum of applications, not only for HPAI but also for other animal health issues.

## Data Availability

All data, except farms characteristics, are publicly available following the different addresses specified in the material and method section. Outbreak-specific data are confidential and cannot be shared publicly. The code for analysis in addition to a fictive dataset are posted on github (https://github.com/MaryemBenS/HPAI-/tree/master/HPAI_code). Requests to access the datasets should be directed to MS, maryem.bensalem@anses.fr.

## Footnotes

^1^ https://www.sandre.eaufrance.fr

^2^ https://geoservices.ign.fr/

^3^ https://www.legifrance.gouv.fr

^4^ https://gadm.org/

## Supplementary material

Supplementary Figure 1 Optimal number of clusters in each scenario: A. Scenario A with farm characteristics and environmental variables; B. Scenario B with only environmental variables.

Supplementary Figure 2 Descriptive statistics for the farm characteristics in the two clusters of scenario A, as obtained by the partitioning around medoids method. For the farm characteristics plots, abbreviations were used as follows: Farm type: commercial (CMMF), backyard (BCKY), and captive (CPTV); Production types: decoy ducks (DCYD), starter phase (STRP), game birds farm (GMBF), fattening (FTTN), future laying hens (FTLH), rearing period (RRNP), laying hens (LYNH), assisted-feeding (GAVA), future breeders (FTRB), breeders (BRDR); Species: Turkey (TRKY), Duck (DUCK), Gallus gallus (GLLG), Multi-species without ducks or geese (MLT-SP CSWITHTW), Multi-species with ducks or geese (MLT-SP CSWITHWT), and other (OTHR).

Supplementary Figure 3 Descriptive statistics for environmental variables in scenario A that addresses water surfaces and roads (Dist. Water Surf., Nb. Water Surf., Water Surf. Cover, Nb. Roads, Road Length and Dist. Roads).

Supplementary Figure 4 Descriptive statistics for environmental variables in scenario B that addresses water surfaces and roads (Dist. Water Surf., Nb. Water Surf., Water Surf. Cover, Nb. Roads, Road Length and Dist. Roads).
