# Supplementary figures and images for: Investigating the role of environmental factors in the French highly pathogenic avian influenza epizootic in 2022–2023
Source: Front Vet Sci. 2025 Jun 13;12:1541019. doi: 10.3389/fvets.2025.1541019 (PMC12202217; doi:10.3389/fvets.2025.1541019)

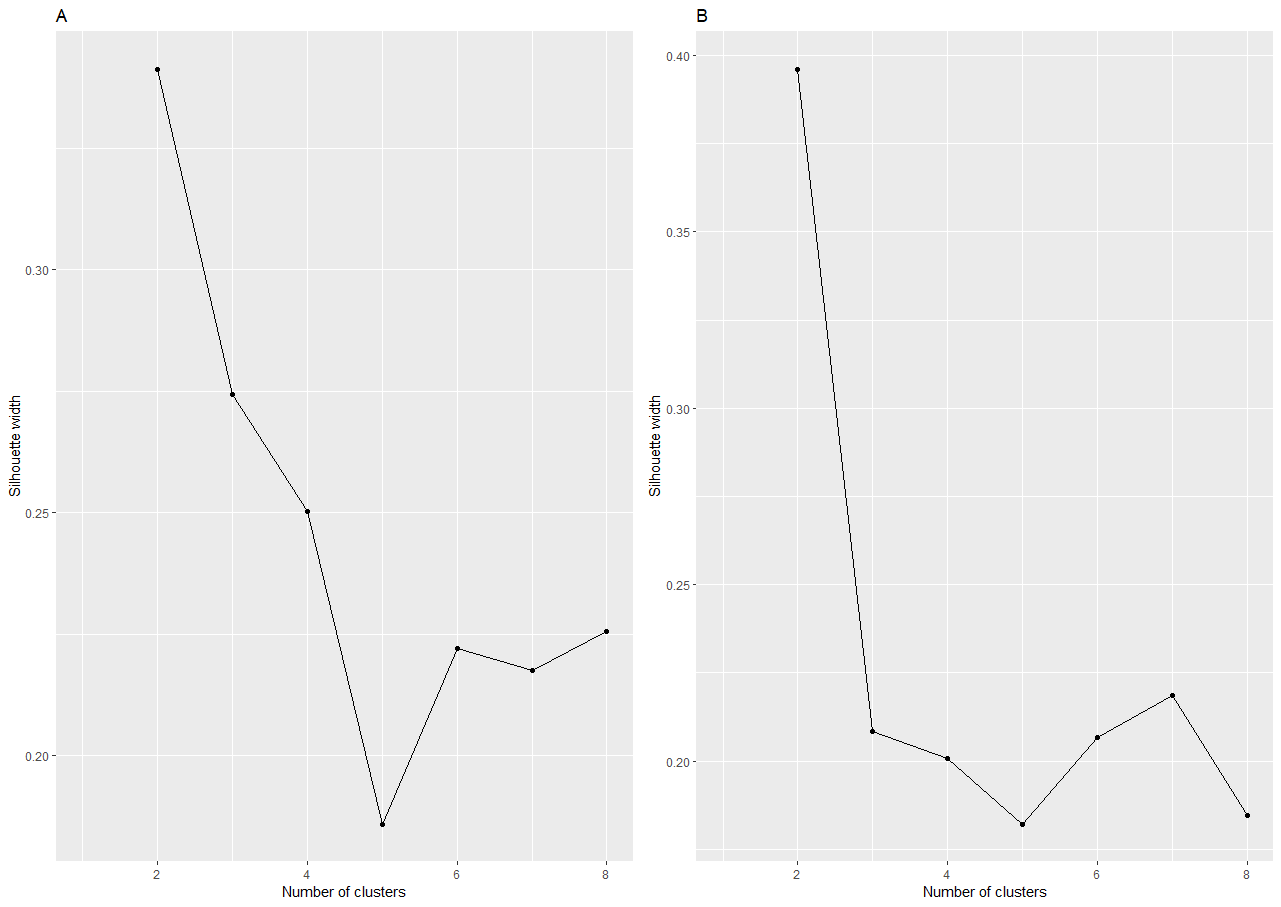

Supplement: Supplementary Figure 1 — Optimal number of clusters in each scenario: A. Scenario A with farm characteristics and environmental variables; B. Scenario B with only environmental variables. [file Image_1.tif]

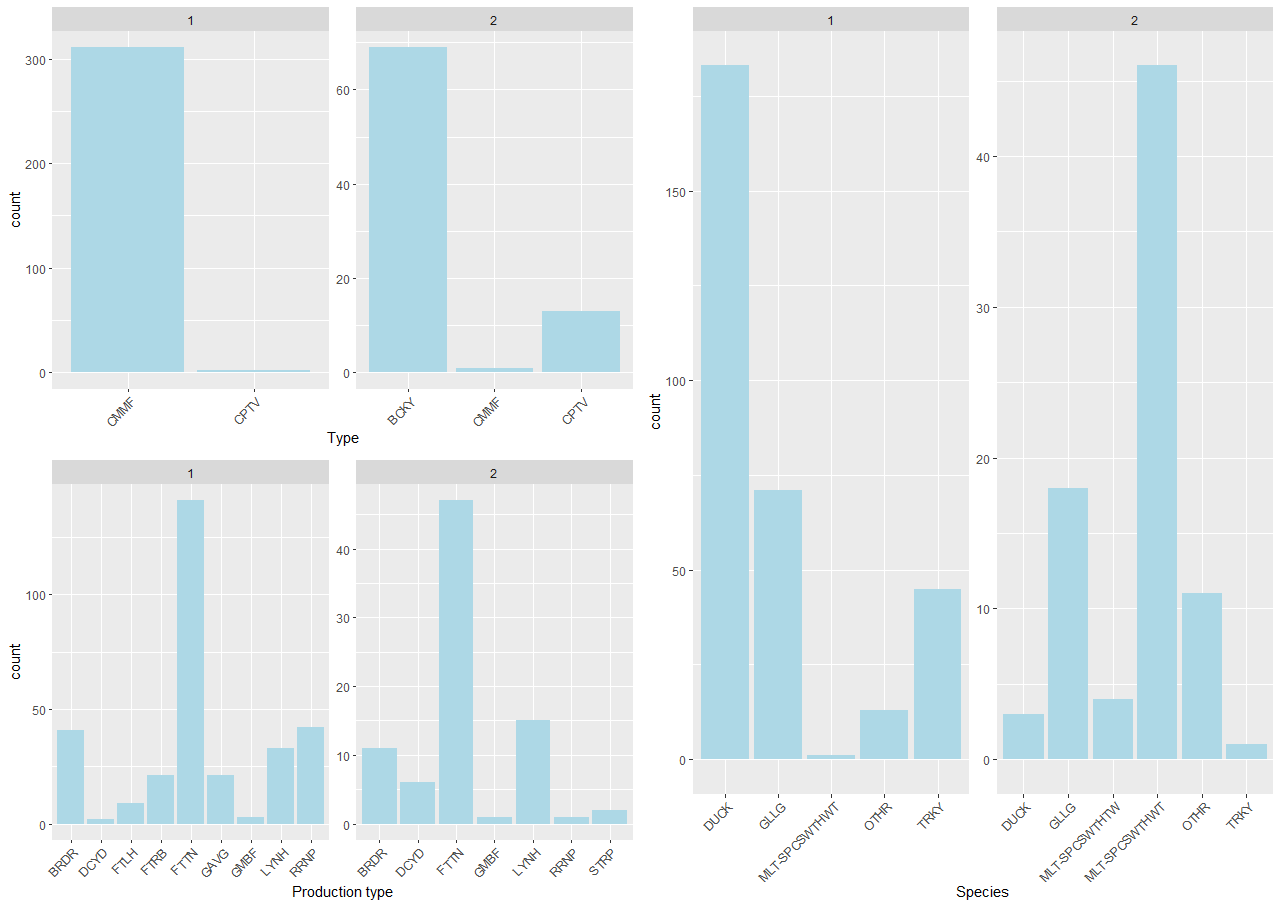

Supplement: Supplementary Figure 2 — Descriptive statistics for the farm characteristics in the two clusters of scenario A, as obtained by the partitioning around medoids method. For the farm characteristics plots, abbreviations were used as follows: Farm type: commercial (CMMF), backyard (BCKY), and captive (CPTV); Production types: decoy ducks (DCYD), starter phase (STRP), game birds farm (GMBF), fattening (FTTN), future laying hens (FTLH), rearing period (RRNP), laying hens (LYNH), assisted-feeding (GAVA), future breeders (FTRB), breeders (BRDR); Species: Turkey (TRKY), Duck (DUCK), Gallus gallus (GLLG), Multi-species without ducks or geese (MLT-SP CSWITHTW), Multi-species with ducks or geese (MLT-SP CSWITHWT), and other (OTHR). [file Image_2.tiff]

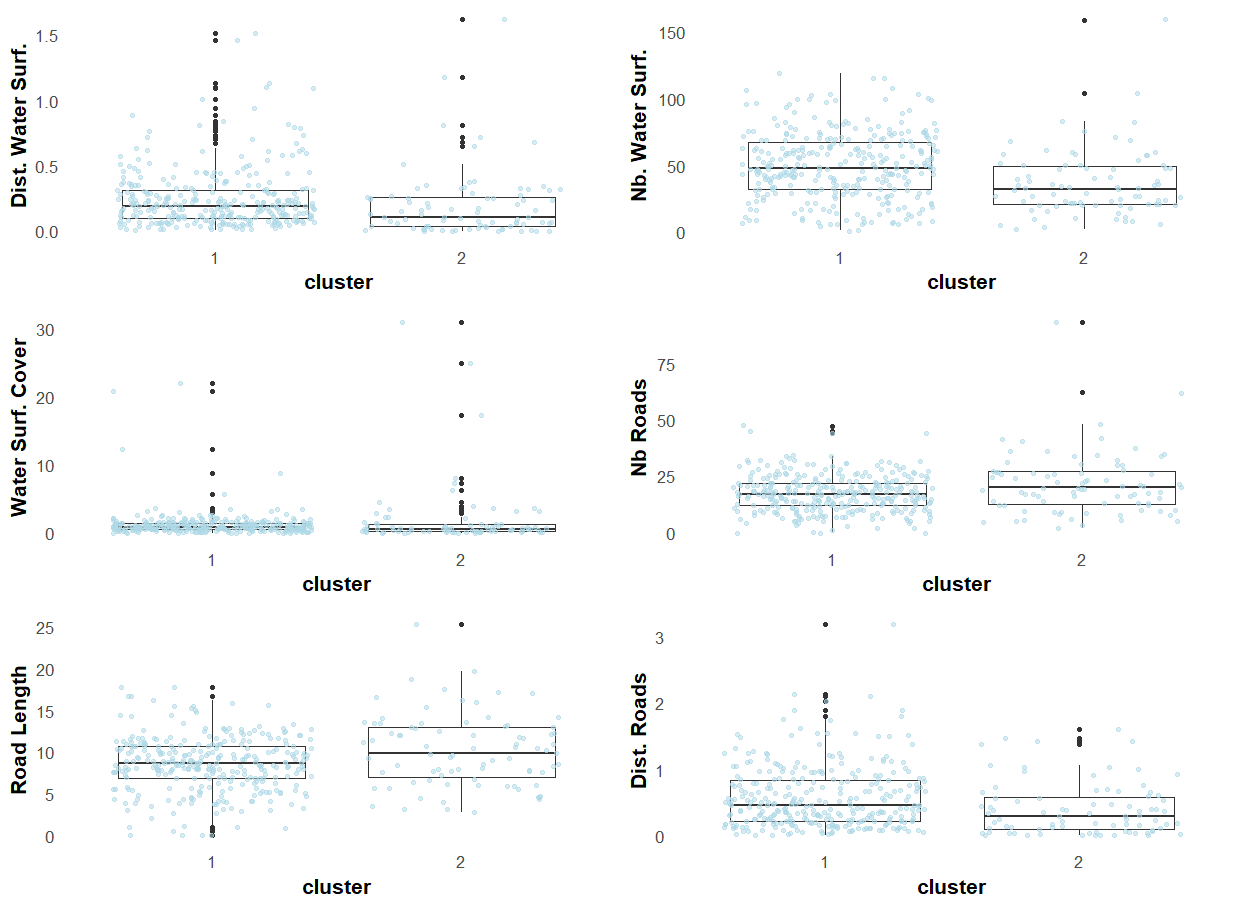

Supplement: Supplementary Figure 3 — Descriptive statistics for environmental variables in scenario A that addresses water surfaces and roads (Dist. Water Surf., Nb. Water Surf., Water Surf. Cover, Nb. Roads, Road Length and Dist. Roads). [file Image_3.tif]

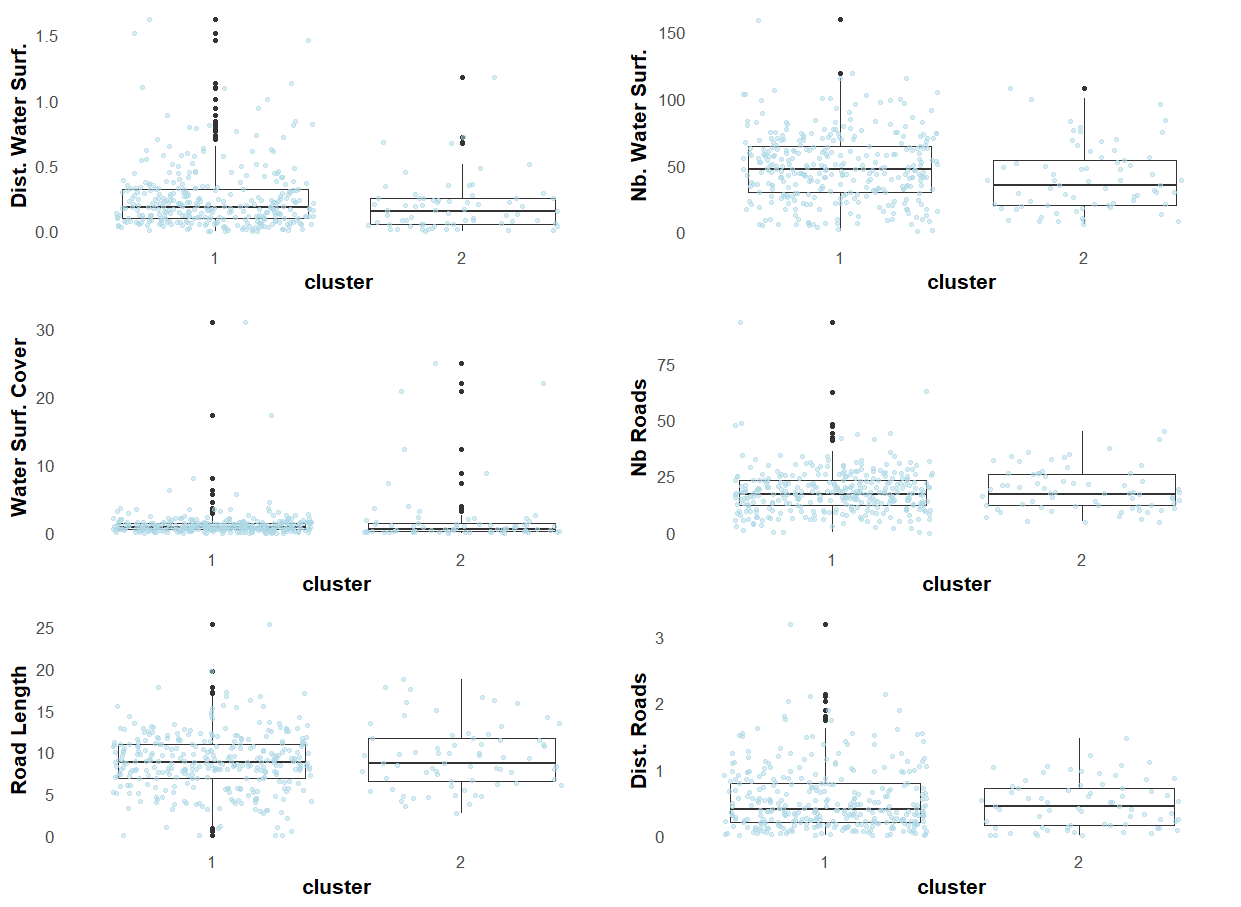

Supplement: Supplementary Figure 4 — Descriptive statistics for environmental variables in scenario B that addresses water surfaces and roads (Dist. Water Surf., Nb. Water Surf., Water Surf. Cover, Nb. Roads, Road Length and Dist. Roads). [file Image_4.tif]
